# Evaluation of artificial intelligence-based autosegmentation for a high-performance cone-beam computed tomography imaging system in the pelvic region

**DOI:** 10.1016/j.phro.2024.100687

**Published:** 2024-12-09

**Authors:** Judith H. Sluijter, Agustinus J.A.J. van de Schoot, Abdelmounaim el Yaakoubi, Maartje de Jong, Martine S. van der Knaap - van Dongen, Britt Kunnen, Nienke D. Sijtsema, Joan J. Penninkhof, Kim C. de Vries, Steven F. Petit, Maarten L.P. Dirkx

**Affiliations:** Department of Radiotherapy, Erasmus MC Cancer Institute, University Medical Center Rotterdam, Rotterdam, The Netherlands

**Keywords:** AI-based autosegmentation, CBCT-guided online adaptive radiotherapy, Pelvic region, Random effects model

## Abstract

**Background and purpose:**

A novel ring-gantry cone-beam computed tomography (CBCT) imaging system shows improved image quality compared to its conventional version, but its effect on autosegmentation is unknown. This study evaluates the impact of this high-performance CBCT on autosegmentation performance, inter-observer variability, contour correction times and delineation confidence, compared to the conventional CBCT.

**Materials and methods:**

Twenty prostate cancer patients were enrolled in this prospective clinical study. Per patient, one pair of high-performance CBCT and conventional CBCT scans was included. Three observers manually corrected contours generated by the artificial intelligence (AI) model for prostate, seminal vesicles, bladder, rectum and bowel. Differences between AI-based and manual corrected contours were quantified using Dice Similarity Coefficient (DSC) and 95th percentile of Hausdorff distance (HD95). Autosegmentation performance and interobserver variation were compared using a random effects model; correction times and confidence scores using a paired *t*-test and Wilcoxon signed-rank test, respectively.

**Results:**

Autosegmentation performance showed small, but statistically insignificant differences. Interobserver variability, assessed by the intraclass correlation coefficient, was significantly different across most organs, but these were considered clinically irrelevant (maximum difference = 0.08). Mean contour correction times were similar for both CBCT systems (11:03 versus 11:12 min; p = 0.66). Delineation confidence scores were significantly higher with the high-performance CBCT scans for prostate, seminal vesicles and rectum (4.5 versus 3.5, 4.3 versus 3.5, 4.8 versus 4.3; all p < 0.001).

**Conclusion:**

The high-performance CBCT did not (clinically) improve autosegmentation performance, inter-observer variability or contour correction time compared to conventional CBCT. However, it clearly enhanced user confidence in organ delineation for prostate, seminal vesicles and rectum.

## Highlights

• The high-performance cone-beam computed tomography (CBCT) did not result in improved autosegmentation performance.
• No clinically relevant improvement in interobserver variability was observed.
• No reduction in contour correction time was achieved.
• The high-performance CBCT enhanced confidence in organ delineation.

## 1 Introduction

Ensuring optimal target coverage and minimal dose to organs at risk (OARs) is challenging for radiotherapy treatment in the pelvic region. Organ motion and variable volumes of the bladder, rectum and bowel may cause daily changes in patient anatomy [1], [2]. Online adaptive radiotherapy (ART) aims to individualize treatments by re-optimizing the treatment plan based on the daily anatomy, thereby accounting for interfractional anatomical variations [3]. Over the last decade, commercially available magnetic resonance (MR)- and cone-beam computed tomography (CBCT)-equipped linear accelerators were introduced to enhance image guidance and enable online ART [4], [5], [6]. CBCT-based online ART has relatively short treatment times, making it suitable for radiotherapy treatment in large patient cohorts [5], [7], [8], [9], [10]. Preliminary studies involving patients with pelvic cancer have shown that online ART may better spare OARs while maintaining target coverage compared to non-adaptive image-guided radiotherapy (IGRT) [8], [10], [11], [12], [13], [14], [15].

For a streamlined online ART workflow with high overall treatment efficiency, integrated artificial intelligence (AI)‐assisted automatic segmentation and treatment planning is required. Currently, CBCT-based online ART typically requires longer timeslots (15–30 min) compared to non-adaptive IGRT (10–12 min) [8], [9], [10], [16]. Minimizing the treatment time in online ART is necessary to enhance patient comfort and efficiently use equipment and staff capacity. Review and adjustment of autosegmentations is the most time-consuming step in online ART workflows, taking typically between 37% and 59% of the total time [8], [10], [11]. Potentially, more accurate autosegmentation models may improve consistency and reduce correction times by requiring fewer adjustments. However, a major reason for suboptimal AI-based autosegmentation is poor CBCT image quality [17], [18], [19].

Recently, a high-performance ring-gantry CBCT imaging system was clinically released, including a newly designed, large imaging panel, fast CBCT acquisition and advanced reconstruction algorithms with metal artefact reduction [6], [20], [21], [22]. While previous studies have demonstrated its improved image quality and reliable Hounsfield unit accuracy for direct dose calculation [21], [22], [23], [24], the impact of this high-performance imaging system on the clinical online ART workflow is not yet quantified. A positive impact might be that the improved image quality translates into increased autosegmentation performance, reduced manual contour adjustments, shorter correction times and/or increased user confidence. Since this high-performance imaging system is associated with considerable additional costs, it is important to identify its added value on clinical workflows.

Therefore, the aim of this study was to evaluate the added value of the high-performance CBCT in the clinical online ART workflow compared to conventional CBCT, focusing on autosegmentation performance, interobserver variability, contour correction times, and delineation confidence.

## 2 Materials and methods

### 2.1 Patient inclusion and imaging

Twenty consecutive prostate cancer patients treated with non-adaptive IGRT between April and October 2023 were included in this prospective study, after providing informed consent (IRB protocol MEC-2022–0815). Patients with anatomical anomalies (prostatectomy (n = 6), transurethral prostate resection (n = 4), extracapsular extension (n = 3), or bladder diverticula (n = 1)) were excluded. According to our standard clinical procedure, all patients except one (due to anticoagulant use) had intra-prostatic gold fiducial markers implanted for position verification purposes. The mean weight of the patients was 84 kg (range: 59–150).

Patients were treated on an Ethos therapy system (Varian Medical Systems, Inc.) equipped with either the high-performance CBCT (HyperSight) or the conventional CBCT imaging (Halcyon 3.1). Daily CBCT imaging was performed for position verification prior to treatment as part of our standard clinical routine. During the treatment course, each patient was treated at least once on consecutive days on an Ethos therapy system with HyperSight CBCT imaging and on an identical system with conventional CBCT imaging. Per patient, one pair of CBCT scans was included, resulting in a total of 40 CBCT scans. All scans were blinded and anonymized for evaluation.

All HyperSight CBCT scans were acquired using the standard acquisition settings for prostate cancer patients with auto-adjusted exposure (mAs) and reconstructed using an iterative algorithm with metal artefact reduction (iCBCT MAR). Conventional CBCT scans were also acquired using their standard acquisition settings, which involved a fixed exposure and reconstruction with a less advanced iterative algorithm (iCBCT). Details on image acquisition and reconstruction parameters are specified in Appendix A.

### 2.2 AI-based contouring workflow

Contours of the prostate, seminal vesicles, bladder, rectum and bowel were autosegmented on all CBCT scans using an integrated AI model in a non-clinical environment identical to our current clinical system (Ethos emulator version 1.1, Varian Medical Systems, Inc.). The organ-specific AI model has a U-Net-based architecture, specifically based on the Tiramisu architecture, and was trained in a supervised learning setting [5], [25]. The training set consisted of 200 to 400 scans, including CBCT and fan-beam CT scans, annotated by anatomy experts from clinics worldwide. The high-performance CBCT scans were not part of the training set.

In the routine clinical online ART workflow, users review AI-generated autosegmented contours and manually them as needed. For this study, three experienced observers, i.e., two radiotherapy technologists (RTTs) and one technical physician [26], [27], independently performed this workflow for five organs on all CBCT scans, resulting in a total of 600 delineations. First, the contours were manually corrected according to our clinical online ART protocol for prostate cancer to determine clinical correction times. According to this protocol, the entire prostate and seminal vesicles were corrected, whereas adjustments for the bladder, rectum, and bowel were limited to a 2 cm wide region around the prostate and seminal vesicles. The delineation confidence of each organ was scored from 1 to 5, where 1 indicated very uncertain and 5 completely confident. The clinical correction time was defined as the time from the start until the end of the adjustments according to our clinical protocol. Next, the observers also manually corrected bladder, rectum and bowel contours outside the 2 cm wide region to allow evaluation of the autosegmentation performance in the entire pelvic region.

### 2.3 Evaluation and statistical analysis

The differences between the AI-based autosegmented and fully corrected contours were quantified using the Dice Similarity Coefficient (DSC) and the 95th percentile Hausdorff distance (HD95) [28], [29]. A random effects model was used to compare the autosegmentation performance when using the high-performance CBCT versus conventional CBCT imaging. This approach allowed for robust estimation of the impact of CBCT type while accounting for variability due to observer and patient heterogeneity. The fixed effect of interest was the CBCT type, categorized as either high-performance CBCT or conventional CBCT. Random intercepts were included for patients and observers to account for the variability within these groups. The significance of the CBCT type coefficient was determined by p-values from Kenward-Roger adjusted standard errors and degrees of freedom [30]. Details of the statistical code and results are provided in Appendix B.

Interobserver variability was assessed using the intraclass correlation coefficient (ICC), which measures consistency among observers on a scale from 0 to 1. In this study, the ICC is defined as the ratio of observer variance to total variance, which includes systematic, patient and observer variance. Note that our definition of ICC differs from the standard approach that typically focuses on minimizing the systematic error with only one random effect [31]. In our study, an ICC close to 0 indicates an observer variance that is small compared to the total variance, indicating high consistency amongst observers. Point estimates were calculated to summarize the interobserver variability based on the sample data. To account for variability and uncertainty in the data, a stratified bootstrapping method with 10,000 permutations was applied to construct confidence intervals. The Welch two sample *t*-test was used to test for statistically significant differences in ICC measures between the high-performance CBCT and conventional CBCT scans. For more details on the statistical approach, see Appendix B.

Clinical correction times and delineation confidence scores were averaged for each CBCT imaging type across the three observers. The significance of differences in contour correction times between the high-performance CBCT and conventional CBCT was assessed using a paired *t*-test, and differences in delineation confidence scores were evaluated using a Wilcoxon signed-rank test.

A p-value of 0.05 was used to assess statistical significance. To account for multiple comparisons, a Bonferroni correction was applied to adjust the significance levels for the 26 tests (10 for autosegmentation performance, 10 for interobserver variability, 1 for contour correction time, and 5 for delineation confidence). This resulted in a corrected significance level of 0.05/26 = 0.0019 per test. Statistical analyses were conducted using R (version 4.4.0) and IBM SPSS Statistics (version 15.0).

## 3 Results

**Fig. 1:**
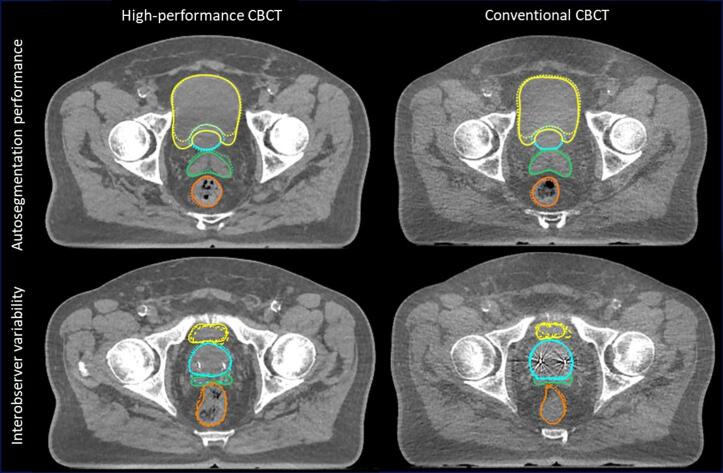
(color in print). Examples of CBCT image quality, autosegmentation performance and interobserver variability for a high-performance CBCT scan (left) and a conventional CBCT scan (right) of the same patient. The structures of the prostate (blue), seminal vesicles (green), bladder (yellow), and rectum (orange) are displayed. The bowel was also segmented, but is not depicted in these examples. Top row: Autosegmentation performance (dotted = artificial intelligence (AI)-based autosegmented contour, solid = fully corrected contour of one observer). Bottom row: interobserver variability (dot-dashed = observer 1, solid = observer 2, dashed = observer 3). Abbreviations: CBCT, Cone-beam computed tomography. (For interpretation of the references to color in this figure legend, the reader is referred to the web version of this article.)

**Fig. 2:**
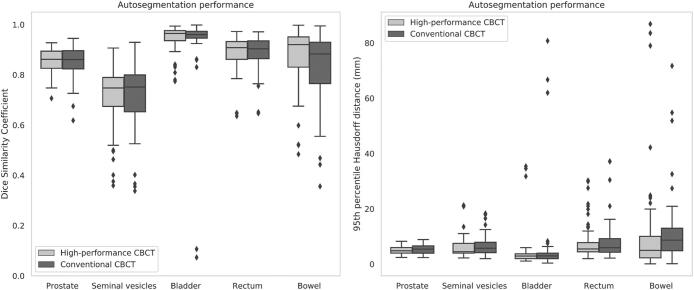
Boxplots representing the autosegmentation performance for the high-performance CBCT and conventional CBCT based on the Dice Similarity Coefficient (DSC) (left) and the 95th percentile of Hausdorff distance (HD95) (right). The boxes represent the interquartile range (IQR), with the 25th (Q1) and 75th (Q3) percentiles. Horizontal lines within the boxes indicate the median values. Outliers are displayed as individual data points and are defined as values below Q1 − 1.5*IQR or above Q3 + 1.5*IQR. Abbreviations: CBCT, Cone-beam computed tomography; mm, millimeter.

Typical examples of CBCT image quality, autosegmentation performance and interobserver variability are shown in Fig. 1. One patient was excluded from bowel comparisons because the bowel was not visible on the high-performance CBCT scan due to a limited cranial field of view. Descriptive statistics for the autosegmentation performance are presented in Table 1 and its results are presented in boxplots in Fig. 2. The random effects model did not demonstrate significant differences in autosegmentation performance between the CBCT types for all organs, as indicated by the coefficients of the CBCT type that are not statistically significant from 0 (Table 2). Note, DSC was similar or higher and HD95 was smaller for all organs with the high-performance CBCT compared to conventional CBCT imaging, but these differences were not significant.

**Table 1:** Autosegmentation performance, evaluated using the Dice Similarity Coefficient and the 95th percentile of the Hausdorff distance in mm between the AI-based autosegmented contour and the manually adjusted contour. Data is presented as mean [standard deviation] for both the high-performance CBCT and conventional CBCT across five organs. Abbreviations: CBCT, Cone-beam computed tomography; mm, millimeter.

	Dice similarity coefficient		95th percentile Hausdorff distance	
	High-performance CBCT	Conventional CBCT	High-performance CBCT	Conventional CBCT
Prostate	0.86 [0.05]	0.85 [0.06]	5.03 [1.38]	5.37 [1.67]
Seminal vesicles	0.71 [0.12]	0.71 [0.13]	6.14 [4.08]	6.76 [4.02]
Bladder	0.95 [0.05]	0.91 [0.19]	4.53 [6.89]	6.49 [14.85]
Rectum	0.89 [0.07]	0.89 [0.07]	8.25 [6.58]	8.38 [7.22]
Bowel	0.86 [0.13]	0.83 [0.13]	11.33 [18.83]	12.11 [13.35]

**Table 2:** Difference between the high-performance CBCT and conventional CBCT resulting from the random effects model to evaluate autosegmentation performance. Note, positive values for Dice Similarity Coefficient (DSC) (left) and negative values for 95th percentile Hausdorff distance (HD95) indicate results that are in favor for the high-performance CBCT. Values are presented as estimated coefficients (standard error). Abbreviations: CBCT, cone-beam computed tomography; mm, millimeter; DSC, Dice Similarity Coefficient; HD95, 95th percentile Hausdorff distance.

	Coefficient for DSC	p-value	Coefficient for HD95	p-value
Prostate	0.00 (0.01)	0.53	−0.35 (0.21)	0.11
Seminal vesicles	−0.00 (0.01)	0.76	−0.63 (0.40)	0.12
Bladder	0.03 (0.02)	0.04	−1.95 (0.85)	0.02
Rectum	−0.00 (0.01)	0.62	−0.13 (0.90)	0.89
Bowel	0.03 (0.02)	0.08	−0.68 (2.76)	0.81

Low DSC scores (<0.2) for bladder and corresponding high HD95 values (>60 mm) on conventional CBCT scans stand out as seen in Fig. 2. These values correspond to one patient, weighing 150 kg, considerably higher than the mean weight of 84 kg. With the high-performance CBCT, image quality was preserved due to auto-adjusted exposure, while the conventional CBCT, acquired with a fixed default exposure, showed poor image quality.

The interobserver variability as quantified by the ICC was small for both CBCT scans (0.07 on average, maximum of 0.22 for all comparisons), indicating that the observers were consistent among each other (Table 3). This low ICC value suggests that the variability in autosegmentation performance is primarily due to differences in the CBCT types and not due to inconsistencies between observers. Both for DSC and for HD95, the difference in ICC for the high-performance and conventional CBCT scans was significant for almost all organs. However, these differences were only minor (maximum 0.08) and were considered clinically irrelevant.

**Table 3:** Interobserver variability evaluated by the intraclass correlation coefficient (ICC), using either point estimates or bootstrap. Stratified bootstrapping (10,000 permutations) was employed to assess if differences in ICC measures between the high-performance CBCT and conventional CBCT were statistically significant. Data shows the ICC (mean ICC for bootstrap) and the difference in the 95% confidence interval (Δ 95% CI). A star sign (*) indicates a significant difference (p-value < 0.0019). Abbreviations: CBCT, Cone-beam computed tomography; ICC, intraclass correlation coefficient; Δ 95% CI, difference in 95% confidence interval; DSC, Dice Similarity Coefficient; HD95, 95th percentile Hausdorff distance.

	ICC point estimate		ICC Bootstrap			
	High-performance CBCT	Conventional CBCT	High-performance CBCT	Conventional CBCT	Δ 95% CI (lower, upper)	p-value
ICC for DSC						
Prostate	0.00	0.00	0.01	0.01	(−0.00, −0.00)	<0.001*
Seminal vesicles	0.11	0.18	0.14	0.22	(0.08, 0.08)	<0.001*
Bladder	0.07	0.00	0.10	0.10	(−0.00, 0.01)	0.25
Rectum	0.09	0.09	0.13	0.15	(0.02, 0.02)	<0.001*
Bowel	0.02	0.00	0.04	0.02	(−0.02, −0.02)	<0.001*

ICC for HD95						
Prostate	0.00	0.00	0.03	0.03	(0.00, 0.01)	<0.001*
Seminal vesicles	0.02	0.04	0.04	0.07	(0.03, 0.04)	<0.001*
Bladder	0.00	0.00	0.06	0.02	(−0.05, −0.04)	<0.001*
Rectum	0.00	0.00	0.01	0.04	(0.03, 0.03)	<0.001*
Bowel	0.01	0.01	0.04	0.04	(0.00, 0.01)	<0.001*

The mean clinical correction time of all five structures combined was 11:03 [3:17] min (mean [standard deviation]) for the high-performance CBCT, versus 11:12 [3:10] min for conventional CBCT imaging (p = 0.66). These differences are clinically negligible, given the total time for one online ART fraction is currently around 25 min in our institute.

The mean delineation confidence score was 4.6 (with 5.0 = completely confident) for high-performance CBCT scans, compared to a score of 4.0 (=confident) for conventional CBCT scans. For prostate, seminal vesicles, and rectum the delineation confidence scores were significantly higher in the high-performance CBCT scans, with no significant differences for bladder and bowel (Table 4).

**Table 4:** Delineation confidence scores for each organ on high-performance CBCT and conventional CBCT, where 1.0 indicates very uncertain and 5.0 completely confident. Data is presented as mean [standard deviation]. A star sign (*) indicates a significant difference (p-value < 0.0019). Abbreviations: CBCT, Cone-beam computed tomography.

	High-performance CBCT	Conventional CBCT	p-value
Prostate	4.5 [0.6]	3.5 [1.0]	<0.001*
Seminal vesicles	4.3 [0.7]	3.5 [1.0]	<0.001*
Bladder	4.9 [0.5]	4.5 [0.9]	0.02
Rectum	4.8 [0.5]	4.3 [1.0]	<0.001*
Bowel	4.6 [0.7]	4.4 [0.9]	0.28

## 4 Discussion

In this study, we evaluated the added value of a novel high-performance ring-gantry CBCT imaging system compared to conventional ring-gantry CBCT in the clinical online ART workflow. For the 5 organs considered, with 120 observations per organ (20 patients, 2 CBCT scans each, 3 observers), we found that the high-performance CBCT did not significantly improve autosegmentation performance and contour correction time, nor did it result in clinically relevant improvements in interobserver variability compared to the conventional CBCT. Consequently, the introduction of the high-performance CBCT did not reduce overall treatment times for online ART. However, it enhanced user confidence in organ delineation for prostate, seminal vesicles and rectum.

In our study, we compared AI-based autosegmentations with manual segmentations derived from AI-based contours to mimic the clinical online ART workflow and ensure that the measures solely reflect relevant corrections. The seminal vesicles demonstrated the lowest DSC values, with a median of 0.71 for both high-performance and conventional CBCT. This can likely be attributed to their small size, where even minor adjustments can largely affect the DSC, while the HD95 values were less sensitive. Similar DSC values of 0.70 for the seminal vesicles have been reported by other studies [32], [33]. For the prostate, bladder, rectum and bowel, we found DSC values ranging from 0.83 to 0.95, similar to values reported in studies using conventional CBCT imaging or CT, reporting DSC values between 0.82 and 0.93 for these organs [32], [34]. These results indicate that the autosegmentation performance is not perfect yet, indicating that review by a human user remains necessary, particularly for target structures and OARs in close proximity to the target structures (typically within 2 cm).

Additionally, our results align with those of Choi et al., who investigated the inter- and intra-observer variability in prostate delineations between CT and conventional C-arm-based CBCT imaging [35]. They found no significant differences in interobserver variability between CT scans with different image quality, which align with our results. Overall, our reported interobserver variability was small, as the ICC results were close to 0. This indicates that, regardless of the CBCT type, the observers were very consistent amongst each other.

Zwart et al. reported a notably shorter mean contour correction time during CBCT-based online ART on a linac equipped with conventional ring gantry CBCT imaging (6:30 min) compared to our mean time (11:03 min) [10]. The inclusion of the bowel structure in our online ART workflow may partly explain this difference. Additionally, as our study was performed in a non-clinical environment, there was less time pressure, although observers were instructed to perform contouring in the same way as in clinical procedures. While the absolute values of the contour correction time may slightly overestimate our clinical contouring times, the differences are meaningful for comparing both CBCT types.

A limitation of our study was that we did not adjust the exposure (mAs) when acquiring conventional CBCT scans, since the image quality was considered sufficient for evaluating patient positioning in our clinical non-adaptive IGRT workflow. Likely, patients with higher weights would have benefited from an adjusted exposure, resulting in improved CBCT image quality and consequently smaller image quality differences compared to high-performance CBCT scans. Moreover, all patients except one had intra-prostatic markers implanted, which locally reduced conventional CBCT image quality due to reconstruction artefacts, as the metal artefact reduction algorithm is not available for this CBCT imaging. We expect that our conclusions also apply for a patient cohort without intra-prostatic makers, as any potential difference between the high-performance CBCT and conventional CBCT would be smaller.

A novel aspect of our study is the introduction of a new statistical framework to evaluate the effect of an explanatory factor (CBCT type) in multi-level data. This approach accounts for patient and observer heterogeneity, enabling accurate assessment of autosegmentation performance and interobserver variability. Traditionally, the most basic and widely used approach to evaluate these metrics is to create a consensus structure by combining observer segmentations [36]. However, the consensus structure approach results in a loss of information and does not provide guidance on how many experts should agree before the structure can be considered valid. Additionally, vote-counting strategies do not consider the potential variability in quality or performance among the observers, as they treat each observer equally [37].

No substantial differences in autosegmentation performance for the male pelvic region were observed when comparing the contours generated by the AI model used in current clinical practice (version 1.1) with those generated by a new preclinical version (version 2.0). Therefore, it is likely that our results remain applicable for the next clinical release of this integrated AI model. Furthermore, using an AI model specifically trained on fan-beam CT and high-performance CBCT scans could improve autosegmentation performance for the high-performance CBCT and potentially reduce online ART treatment times.

Considering the high autosegmentation performance, the question arises whether manual correction might result in statistically significant differences in dose metrics. Other studies have already shown no statistically significant differences in dose metrics when comparing doses from automated and manually delineated contours of bladder and rectum in patients with prostate cancer [38], [39]. To evaluate potential timesaving benefits in patient treatment, further research on the dosimetric impact of manual corrections is required.

The improved delineation confidence is a clear advantage of the high-performance CBCT, since it might accelerate the training of RTTs for performing online ART workflows without supervision (RTT-only workflow), as higher confidence levels have shown to be related to an improved learning performance [40]. Additionally, confidence, in combination with competence, is described as an important factor for a safe and efficient RTT-only workflow [41], [42]. This potential acceleration is relevant given current staff shortages [43], as a RTT-only workflow reduces the need for supervision by radiation oncologists and/or medical physicists during online ART.

In conclusion, although the high-performance CBCT demonstrates improved image quality, it did not lead to (clinically) significant improvements in autosegmentation performance, overall interobserver variability and contour correction time in the male pelvic region compared to conventional ring gantry CBCT imaging. However, it clearly enhanced user confidence in organ delineation for prostate, seminal vesicles and rectum.

## CRediT authorship contribution statement

**Judith H. Sluijter:** Conceptualization, Methodology, Software, Validation, Formal analysis, Investigation, Data curation, Writing – original draft, Writing – review & editing, Visualization, Supervision, Project administration. **Agustinus J.A.J. van de Schoot:** Conceptualization, Methodology, Validation, Writing – review & editing. **Abdelmounaim el Yaakoubi:** Methodology, Software, Validation, Formal analysis, Data curation, Writing – review & editing. **Maartje de Jong:** Methodology, Investigation, Writing – review & editing. **Martine S. van der Knaap - van Dongen:** Methodology, Investigation, Writing – review & editing. **Britt Kunnen:** Conceptualization, Methodology, Validation, Resources, Writing – review & editing. **Nienke D. Sijtsema:** Conceptualization, Methodology, Validation, Writing – review & editing. **Joan J. Penninkhof:** Conceptualization, Methodology, Validation, Writing – review & editing. **Kim C. de Vries:** Conceptualization, Methodology, Writing – review & editing. **Steven F. Petit:** Conceptualization, Methodology, Validation, Writing – review & editing. **Maarten L.P. Dirkx:** Conceptualization, Methodology, Validation, Writing – review & editing, Supervision.

## Declaration of competing interest

The authors declare the following financial interests/personal relationships which may be considered as potential competing interests: Erasmus MC Cancer Institute has research collaborations with Varian, a Siemens Healthineers Company (Palo Alto, CA, USA), Elekta AB (Stockholm, Sweden) and Accuray (Sunnyvale, CA, USA). Varian was not involved in this study and had no role in study design, data collection and analysis, and decisions on preparation of the manuscript. None of the authors has any affiliation with Varian. There are no other conflicts of interests to declare from all authors. No funding was received for this study.
