# Supplementary material for: Evaluation of artificial intelligence-based autosegmentation for a high-performance cone-beam computed tomography imaging system in the pelvic region
Source: Phys Imaging Radiat Oncol. 2024 Dec 9;33:100687. doi: 10.1016/j.phro.2024.100687 (PMC11721864; doi:10.1016/j.phro.2024.100687)
Supplement: Supplementary Data 1 [file mmc1.pdf]

Appendix A. Details on image acquisition and reconstruction

Table A1. *Details on image acquisition and reconstruction for the high-performance CBCT and conventional CBCT. Abbreviations: CBCT, Cone-beam computed tomography; kVp, kilovoltage peak; s, seconds; mAs, milliampere-seconds; CTDIvol, CT dose index; mGy, milligray; mm, millimeter.*

	**High-performance CBCT (n=20)**	**Conventional CBCT (n=20)**	
**Acquisition mode**	Pelvis (n=20)	Pelvis Fast (n= 17)	Pelvis (n = 3)
**kVp**	125	125	125
**Scan time [s]**	5.9	21.2	36.7
**Exposure [mAs] (median)**	529 (range: 345-915)	591	1072
**CTDIvol [mGy] (median)**	10.1 (range: 6.6-17.4)	11.8	21.5
**Reconstruction**	iCBCT MAR	iCBCT	
**In-plane resolution [mm]**	1.05\1.05	0.96\0.96	
**Slice thickness [mm]**	2.0	2.0	
**Reconstruction diameter [mm]**	538	492	

Appendix B

Statistical Analysis Report: Evaluation of artificial intelligence-based autosegmentation for a high-performance cone-beam computed tomography imaging system in the pelvic region

Author: Abdelmounaim el Yaakoubi

22 July, 2024

Contents

Contour Correction Times **2**

Confidence Scores **4**

Random Effect model **6**

Model Selection 6

Dice Similarity Coefficient 7

Effect CBCT type on Autosegmentation performance 7

ICC: Intra Class Correlation 13

Bootstrapping procedure 25

Hausdorff Distance 28

Effect CBCT type on Autosegmentation performance 28

ICC: Intra Class Correlation 33

Bootstrapping procedure 44

Note 47

Contour Correction Times

The Jarque-Bera test is performed to test for normality. If the null hypothesis for normality is not rejected the t-test is performed, otherwise a non-parametric approach is chosen.

```
library(tsoutliers)
sapply(data_sec, function(x) c(
                         "Mean"= mean(x,na.rm=TRUE),
                         "Standard deviation" = sd(x, na.rm=TRUE),
                         "n" = length(x),
                         "Median" = median(x,na.rm=TRUE),
                         "Minimum" = min(x,na.rm=TRUE),
                         "Maximun" = max(x,na.rm=TRUE)
))
```

```
                   all_cCBCt    all_HS
Mean                672.8167  663.1333
Standard deviation  190.8463  197.2538
n                    60.0000   60.0000
Median              624.5000  632.5000
Minimum             399.0000  387.0000
Maximun            1197.0000 1403.0000
```

```
JarqueBera.test(data_sec$all_HS - data_sec$all_cCBCt)
```

```

    Jarque Bera Test

data:  data_sec$all_HS - data_sec$all_cCBCt
X-squared = 4.7091, df = 2, p-value = 0.09494


    Skewness

data:  data_sec$all_HS - data_sec$all_cCBCt
statistic = 0.40327, p-value = 0.2022


    Kurtosis

data:  data_sec$all_HS - data_sec$all_cCBCt
statistic = 4.1105, p-value = 0.07912
```

```
t.test(data_sec$all_HS, data_sec$all_cCBCt, paired = TRUE, alternative = "two.sided")
```

```

    Paired t-test

data:  data_sec$all_HS and data_sec$all_cCBCt
t = -0.43563, df = 59, p-value = 0.6647
alternative hypothesis: true mean difference is not equal to 0
95 percent confidence interval:
```

```
 -54.16262  34.79596
sample estimates:
mean difference
      -9.683333
```

The Jarque-Bera test provides sufficient evidence that the normality assumption holds. Therefore, the t-test is performed, and it shows that there is no significant evidence to show that the high-performance cone-beam CT (HyperSight CBCT = HS) provides faster contour correction times than the conventional CBCT (cCBCT) on a 1.25% significance level.

Confidence Scores

```
sapply(data_conf[,1:5], function(x) c(
                         "Mean"= mean(x,na.rm=TRUE),
                         "Standard deviation" = sd(x, na.rm=TRUE),
                         "n" = length(x),
                         "Median" = median(x,na.rm=TRUE),
                         "Minimum" = min(x,na.rm=TRUE),
                         "Maximun" = max(x,na.rm=TRUE)
)
)
```

```
                   Prostaat_obs1_HS Vesicles_obs1_HS Blaas_obs1_HS
Mean                      4.5166667        4.3333333     4.8500000
Standard deviation        0.6241378        0.7287479     0.5150284
n                        60.0000000       60.0000000    60.0000000
Median                    5.0000000        4.0000000     5.0000000
Minimum                   3.0000000        2.0000000     2.0000000
Maximun                   5.0000000        5.0000000     5.0000000
                   Rectum_obs1_HS Sigmoid_obs1_HS
Mean                    4.7500000       4.6111111
Standard deviation      0.5084039       0.6845096
n                      60.0000000      60.0000000
Median                  5.0000000       5.0000000
Minimum                 3.0000000       3.0000000
Maximun                 5.0000000       5.0000000
```

```
sapply(data_conf[,6:10], function(x) c(
                         "Mean"= mean(x,na.rm=TRUE),
                         "Standard deviation" = sd(x, na.rm=TRUE),
                         "n" = length(x),
                         "Median" = median(x,na.rm=TRUE),
                         "Minimum" = min(x,na.rm=TRUE),
                         "Maximun" = max(x,na.rm=TRUE)
)
)
```

```
                   Prostaat_obs1_cCBCT Vesicles_obs1_cCBCT Blaas_obs1_cCBCT
Mean                          3.450000            3.500000         4.533333
Standard deviation            1.015557            1.033342         0.891897
n                            60.000000           60.000000        60.000000
Median                        3.500000            3.500000         5.000000
Minimum                       1.000000            1.000000         1.000000
Maximun                       5.000000            5.000000         5.000000
                   Rectum_obs1_cCBCT Sigmoid_obs1_cCBCT
Mean                       4.2666667          4.4000000
Standard deviation         0.9719222          0.9349193
n                         60.0000000         60.0000000
Median                     5.0000000          5.0000000
Minimum                    1.0000000          2.0000000
Maximun                    5.0000000          5.0000000
```

Because the scores are ordinal, the two-sided Wilcoxon Signed Rank test is applied to test the high-performance CBCT (HS) against the conventional CBCT (cCBCT).

```
library(exactRankTests)

wilcox.exact(data_conf$Prostaat_obs1_HS, data_conf$Prostaat_obs1_cCBCT, paired = TRUE, alternative = "tw
```

```
	Exact Wilcoxon signed rank test

data:  data_conf$Prostaat_obs1_HS and data_conf$Prostaat_obs1_cCBCT
V = 1009.5, p-value = 3.057e-09
alternative hypothesis: true mu is not equal to 0
```

```
wilcox.exact(data_conf$Vesicles_obs1_HS, data_conf$Vesicles_obs1_cCBCT, paired = TRUE, alternative = "tw
```

```
	Exact Wilcoxon signed rank test

data:  data_conf$Vesicles_obs1_HS and data_conf$Vesicles_obs1_cCBCT
V = 868.5, p-value = 1.338e-06
alternative hypothesis: true mu is not equal to 0
```

```
wilcox.exact(data_conf$Blaas_obs1_HS, data_conf$Blaas_obs1_cCBCT, paired = TRUE, alternative = "two.side
```

```
	Exact Wilcoxon signed rank test

data:  data_conf$Blaas_obs1_HS and data_conf$Blaas_obs1_cCBCT
V = 166, p-value = 0.01757
alternative hypothesis: true mu is not equal to 0
```

```
wilcox.exact(data_conf$Rectum_obs1_HS, data_conf$Rectum_obs1_cCBCT, paired = TRUE, alternative = "two.s
```

```
	Exact Wilcoxon signed rank test

data:  data_conf$Rectum_obs1_HS and data_conf$Rectum_obs1_cCBCT
V = 311, p-value = 0.0001543
alternative hypothesis: true mu is not equal to 0
```

```
wilcox.exact(data_conf$Sigmoid_obs1_HS, data_conf$Sigmoid_obs1_cCBCT, paired = TRUE, alternative = "two
```

```
	Exact Wilcoxon signed rank test

data:  data_conf$Sigmoid_obs1_HS and data_conf$Sigmoid_obs1_cCBCT
V = 249.5, p-value = 0.2846
alternative hypothesis: true mu is not equal to 0
```

Based on the results there is sufficient evidence that the high-performance CBCT (HS) improves Confidence Scores for the Prostate, Seminal Vesicle and, Rectum. For the Bladder and Sigmoid Colon there is no sufficient evidence to reject the null hypothesis based on a 0.25% significance level

Random Effects model

Model Selection

In order to compare the high-performance CBCT (HS) against the conventional CBCT (cCBCT) a Random Effects model is applied in order to take advantage of the longitudinal structure of this data set. There are two outcome parameters that are assessed, one is the Dice coefficient (dice), and the other is the 95th percentile Hausdorff distance (vsdp). The assumption for this analysis is that there is patient heterogeneity, observer heterogeneity and systematic measurements error that cause variance in the primary endpoint, and the type of CBCT will be considered as a explanatory factor. After model-fit, parameter interpretation can be performed, a significance test for the comparison between scan type with corresponding p-value and the point-estimate of the ICC can be calculated.

In statistics, the intraclass correlation, or the intraclass correlation coefficient (ICC) is a descriptive statistic that can be used when quantitative measurements are made on units that are organized into groups. It describes how strongly units in the same group resemble each other. A low ICC in the context of this research indicates good agreement between observers because the observer variance is small relative to the total variance.

Dice Similarity Coefficient

Effect CBCT type on Autosegmentation performance

In the code below, we compare original model, a model with both random effects for patients and observers, with a nested model with only a random effect for the observers. This comparison is useful for model-selection because it quantifies the effect of patient heterogeneity. Formal statistical comparison is made by means of a likelihood ratio test.

```
library(lme4)
library(jtools)

for(organi in unique(data_hs$organ)){

#Print title
cat('\n')
cat(paste(organi, '---------------------------------------------- \n'))
cat('\n')

#Fit the regression model with random effects for the observers and patients
fm = lmer(dice ~ typeCBCT  + (1 | obs) + (1 | ptID) , data = data_hs, subset = organ == organi)
print(summ(fm, digits = 5))

#Fit the regression model with random effects only for the observers
nm = lmer(dice ~ typeCBCT  + (1 | obs) , data = data_hs, subset = organ == organi)


# Compare most suitable model with the Likelihood-ratio test
library(lmtest)
print(lrtest(nm, fm))
#cat('\n')
cat(paste('BIC statistic for the original model : ', round(BIC(fm)), "\n"))
cat(paste('BIC statistic for the nested model : ', round(BIC(nm)), "\n"))
}
```

```
Bladder ----------------------------------------------

MODEL INFO:
Observations: 120
Dependent Variable: dice
Type: Mixed effects linear regression

MODEL FIT:
AIC = -179.33749, BIC = -165.40003
Pseudo-R² (fixed effects) = 0.01297
Pseudo-R² (total) = 0.63440

FIXED EFFECTS:
---------------------------------------------------------------------
                        Est.       S.E.      t val.        d.f.         p
------------------ --------- ---------- ----------- ---------- ---------
(Intercept)          0.91382    0.02750    33.22725    20.95722   0.00000
typeCBCTHS           0.03235    0.01575     2.05441    97.00000   0.04263
```

```
----------------------------------------------------------------------

p values calculated using Kenward-Roger standard errors and d.f.

RANDOM EFFECTS:
------------------------------------
  Group      Parameter    Std. Dev.
---------- ------------- -----------
   ptID     (Intercept)    0.11246
   obs      (Intercept)    0.00002
 Residual                  0.08626
------------------------------------

Grouping variables:
----------------------------
 Group   # groups     ICC
------- ---------- ---------
 ptID       20      0.62960
  obs       3       0.00000
----------------------------
Likelihood ratio test

Model 1: dice ~ typeCBCT + (1 | obs)
Model 2: dice ~ typeCBCT + (1 | obs) + (1 | ptID)
  #Df LogLik Df  Chisq Pr(>Chisq)
1   4 60.295
2   5 94.669  1 68.748  < 2.2e-16 ***
---
Signif. codes:  0 '***' 0.001 '**' 0.01 '*' 0.05 '.' 0.1 ' ' 1
BIC statistic for the original model :  -165
BIC statistic for the nested model :  -101

Seminalvesicle ---------------------------------------------

MODEL INFO:
Observations: 120
Dependent Variable: dice
Type: Mixed effects linear regression

MODEL FIT:
AIC = -224.68371, BIC = -210.74626
Pseudo-R² (fixed effects) = 0.00020
Pseudo-R² (total) = 0.73737

FIXED EFFECTS:
----------------------------------------------------------------------
                        Est.      S.E.     t val.       d.f.         p
----------------- ---------- --------- ---------- ---------- ---------
(Intercept)          0.71478   0.03811   18.75812    5.05280   0.00001
typeCBCTHS          -0.00376   0.01239   -0.30317   97.00000   0.76241
----------------------------------------------------------------------

p values calculated using Kenward-Roger standard errors and d.f.
```

```
RANDOM EFFECTS:
------------------------------------
  Group      Parameter    Std. Dev.
---------- ------------- -----------
   ptID     (Intercept)    0.10180
   obs      (Intercept)    0.05071
 Residual                  0.06788
------------------------------------

Grouping variables:
----------------------------
 Group   # groups     ICC
------- ---------- ---------
 ptID       20      0.59073
  obs       3       0.14659
----------------------------
Likelihood ratio test

Model 1: dice ~ typeCBCT + (1 | obs)
Model 2: dice ~ typeCBCT + (1 | obs) + (1 | ptID)
  #Df  LogLik Df  Chisq Pr(>Chisq)
1   4  75.098
2   5 117.342  1 84.488  < 2.2e-16 ***
---
Signif. codes:  0 '***' 0.001 '**' 0.01 '*' 0.05 '.' 0.1 ' ' 1
BIC statistic for the original model :  -211
BIC statistic for the nested model :  -131

Sigmoidcolon -------------------------------------------------

MODEL INFO:
Observations: 113
Dependent Variable: dice
Type: Mixed effects linear regression

MODEL FIT:
AIC = -154.40235, BIC = -140.76541
Pseudo-R² (fixed effects) = 0.01411
Pseudo-R² (total) = 0.48610

FIXED EFFECTS:
--------------------------------------------------------------------
                       Est.      S.E.     t val.       d.f.         p
----------------- ---------- ---------- ----------- ---------- ---------
(Intercept)          0.83291    0.02462    33.82841   19.84247   0.00000
typeCBCTHS           0.03155    0.01801     1.75174   91.09416   0.08318
--------------------------------------------------------------------

p values calculated using Kenward-Roger standard errors and d.f.

RANDOM EFFECTS:
------------------------------------
  Group      Parameter    Std. Dev.
---------- ------------- -----------
```

```
   ptID     (Intercept)    0.09167
   obs      (Intercept)    0.00001
 Residual                  0.09565
------------------------------------

Grouping variables:
----------------------------
 Group   # groups     ICC
------- ---------- ---------
 ptID       19      0.47875
  obs       3       0.00000
----------------------------
Likelihood ratio test

Model 1: dice ~ typeCBCT + (1 | obs)
Model 2: dice ~ typeCBCT + (1 | obs) + (1 | ptID)
  #Df LogLik Df Chisq Pr(>Chisq)
1   4 63.756
2   5 82.201  1 36.89   1.25e-09 ***
---
Signif. codes:  0 '***' 0.001 '**' 0.01 '*' 0.05 '.' 0.1 ' ' 1
BIC statistic for the original model :  -141
BIC statistic for the nested model :  -109

Prostate ---------------------------------------------

MODEL INFO:
Observations: 120
Dependent Variable: dice
Type: Mixed effects linear regression

MODEL FIT:
AIC = -400.60158, BIC = -386.66412
Pseudo-R² (fixed effects) = 0.00125
Pseudo-R² (total) = 0.62543

FIXED EFFECTS:
---------------------------------------------------------------------
                       Est.      S.E.     t val.       d.f.         p
----------------- --------- --------- ---------- ---------- ---------
(Intercept)         0.85235   0.01070   79.68729   20.96558   0.00000
typeCBCTHS          0.00389   0.00618    0.63066   97.00000   0.52975
---------------------------------------------------------------------

p values calculated using Kenward-Roger standard errors and d.f.

RANDOM EFFECTS:
------------------------------------
  Group      Parameter    Std. Dev.
---------- ------------- -----------
   ptID     (Intercept)    0.04367
   obs      (Intercept)    0.00000
 Residual                  0.03383
------------------------------------
```

```
Grouping variables:
----------------------------
 Group   # groups     ICC
------- ---------- ---------
 ptID       20      0.62496
  obs       3       0.00000
----------------------------
Likelihood ratio test

Model 1: dice ~ typeCBCT + (1 | obs)
Model 2: dice ~ typeCBCT + (1 | obs) + (1 | ptID)
  #Df LogLik Df  Chisq Pr(>Chisq)
1   4 171.48
2   5 205.30  1 67.643  < 2.2e-16 ***
---
Signif. codes:  0 '***' 0.001 '**' 0.01 '*' 0.05 '.' 0.1 ' ' 1
BIC statistic for the original model :  -387
BIC statistic for the nested model :  -324

Rectum ---------------------------------------------

MODEL INFO:
Observations: 120
Dependent Variable: dice
Type: Mixed effects linear regression

MODEL FIT:
AIC = -344.82806, BIC = -330.89060
Pseudo-R² (fixed effects) = 0.00071
Pseudo-R² (total) = 0.64946

FIXED EFFECTS:
----------------------------------------------------------------------
                        Est.      S.E.     t val.       d.f.         p
----------------- ---------- --------- ---------- ---------- ---------
(Intercept)          0.89115   0.01795   49.63540    6.88467   0.00000
typeCBCTHS          -0.00377   0.00769   -0.49054   97.00000   0.62486
----------------------------------------------------------------------

p values calculated using Kenward-Roger standard errors and d.f.

RANDOM EFFECTS:
------------------------------------
  Group      Parameter    Std. Dev.
---------- ------------- -----------
   ptID     (Intercept)    0.05318
   obs      (Intercept)    0.02131
 Residual                  0.04211
------------------------------------

Grouping variables:
----------------------------
 Group   # groups     ICC
```

```
------- ---------- ---------
 ptID       20      0.55936
  obs       3       0.08986
----------------------------
Likelihood ratio test

Model 1: dice ~ typeCBCT + (1 | obs)
Model 2: dice ~ typeCBCT + (1 | obs) + (1 | ptID)
  #Df LogLik Df  Chisq Pr(>Chisq)
1   4 145.13
2   5 177.41  1 64.564  9.344e-16 ***
---
Signif. codes:  0 '***' 0.001 '**' 0.01 '*' 0.05 '.' 0.1 ' ' 1
BIC statistic for the original model :  -331
BIC statistic for the nested model :  -271
```

The results evidently suggest that a model with random effects for patients and observers is more precise, as shown by the statistically significant p-values from the LR-test and the Bayesian Information Criterion. The t-values demonstrate no significant results in favor of the HS on a significance level of 0.125%.

ICC: Intra Class Correlation

In our model there a three sources of variances: the systematic error, the patients heterogeneity, and the observers subjectivity. All three sources contribute to the total variance of the outcome. In an ideal scenario, there firstly would be no observer subjectivity, secondly, the observers would be consistent between each other, and thirdly, the observers would have the same degree of variance between the scans. All three of the above mentioned is practically not the case, and that is why it is of utmost importance to correct for this subjectivity with a statistical model.

The statistical framework I propose in this rapport captures observer heterogeneity by including a random intercept, the variance of this intercept will be used to quantify the degree of consistency between the observers, via the ICC, and lastly, the data will be split between the HS and cCBCT, in order to assess their ICC separately and test them against each other utilizing a Bootstrap method.

```
for(organi in unique(data_hs$organ)){



  #Print title
  cat('\n')
  cat(paste(organi, '-------------------------------------------- \n'))
  cat('\n')

  #Calculate ICC for full model
  data_hsi = subset(data_hs, organ == organi)
  fm = lmer(dice ~ typeCBCT  + (1 | obs) + (1 | ptID) , data = data_hsi)
  print(summ(fm, digits = 5))

  #Calculate ICC for Hypersight records
  cat('\n')
  fm = lmer(dice ~ (1 | obs) + (1 | ptID) , data = data_hsi, subset = typeCBCT == "HS")
  print(summ(fm, digits = 5))

  #Calculate ICC for cCBCT records
  cat('\n')
  fm = lmer(dice ~ (1 | obs) + (1 | ptID) , data = data_hsi, subset = typeCBCT == "cCBCT")
  print(summ(fm, digits = 5))

}
```

```
Bladder --------------------------------------------

MODEL INFO:
Observations: 120
Dependent Variable: dice
Type: Mixed effects linear regression

MODEL FIT:
AIC = -179.33749, BIC = -165.40003
Pseudo-R² (fixed effects) = 0.01297
Pseudo-R² (total) = 0.63440

FIXED EFFECTS:
```

	Est.	S.E.	t val.	d.f.	p
(Intercept)	0.91382	0.02750	33.22725	20.95722	0.00000
typeCBCTHS	0.03235	0.01575	2.05441	97.00000	0.04263

p values calculated using Kenward-Roger standard errors and d.f.

RANDOM EFFECTS:

Group	Parameter	Std. Dev.
ptID	(Intercept)	0.11246
obs	(Intercept)	0.00002
Residual		0.08626

Grouping variables:

Group	# groups	ICC
ptID	20	0.62960
obs	3	0.00000

MODEL INFO:
Observations: 60
Dependent Variable: dice
Type: Mixed effects linear regression

MODEL FIT:
AIC = -279.84712, BIC = -271.46975
Pseudo-R^2 (fixed effects) = 0.00000
Pseudo-R^2 (total) = 0.96721

FIXED EFFECTS:

	Est.	S.E.	t val.	d.f.	p
(Intercept)	0.94618	0.01352	69.95847	12.32038	0.00000

p values calculated using Kenward-Roger standard errors and d.f.

RANDOM EFFECTS:

Group	Parameter	Std. Dev.
ptID	(Intercept)	0.04916
obs	(Intercept)	0.01349
Residual		0.00939

```
Grouping variables:
-----------------------------
 Group   # groups     ICC
------- ---------- ---------
 ptID       20      0.89948
  obs       3       0.06772
-----------------------------

MODEL INFO:
Observations: 60
Dependent Variable: dice
Type: Mixed effects linear regression

MODEL FIT:
AIC = -225.34775, BIC = -216.97037
Pseudo-R² (fixed effects) = 0.00000
Pseudo-R² (total) = 0.99753

FIXED EFFECTS:
---------------------------------------------------------------------
                       Est.      S.E.     t val.       d.f.         p
----------------- ---------- ---------- ----------- ----------- ---------
(Intercept)         0.91382   0.04417   20.69020   19.97783   0.00000
---------------------------------------------------------------------

p values calculated using Kenward-Roger standard errors and d.f.

RANDOM EFFECTS:
------------------------------------
  Group      Parameter    Std. Dev.
---------- ------------- -----------
   ptID     (Intercept)    0.19454
   obs      (Intercept)    0.01306
 Residual                  0.00971
------------------------------------

Grouping variables:
-----------------------------
 Group   # groups     ICC
------- ---------- ---------
 ptID       20      0.99305
  obs       3       0.00448
-----------------------------

Seminalvesicle ---------------------------------------------

MODEL INFO:
Observations: 120
Dependent Variable: dice
Type: Mixed effects linear regression

MODEL FIT:
AIC = -224.68371, BIC = -210.74626
Pseudo-R² (fixed effects) = 0.00020
```

Pseudo-R^2 (total) = 0.73737

FIXED EFFECTS:

	Est.	S.E.	t val.	d.f.	p
(Intercept)	0.71478	0.03811	18.75812	5.05280	0.00001
typeCBCTHS	-0.00376	0.01239	-0.30317	97.00000	0.76241

p values calculated using Kenward-Roger standard errors and d.f.

RANDOM EFFECTS:

Group	Parameter	Std. Dev.
ptID	(Intercept)	0.10180
obs	(Intercept)	0.05071
Residual		0.06788

Grouping variables:

Group	# groups	ICC
ptID	20	0.59073
obs	3	0.14659

MODEL INFO:
Observations: 60
Dependent Variable: dice
Type: Mixed effects linear regression

MODEL FIT:
AIC = -123.16500, BIC = -114.78762
Pseudo-R^2 (fixed effects) = 0.00000
Pseudo-R^2 (total) = 0.87128

FIXED EFFECTS:

	Est.	S.E.	t val.	d.f.	p
(Intercept)	0.71103	0.03554	20.00483	6.85393	0.00000

p values calculated using Kenward-Roger standard errors and d.f.

RANDOM EFFECTS:

Group	Parameter	Std. Dev.
ptID	(Intercept)	0.11084
obs	(Intercept)	0.04293

```
 Residual                  0.04569
------------------------------------

Grouping variables:
----------------------------
 Group   # groups     ICC
------- ---------- ---------
 ptID       20      0.75762
  obs       3       0.11365
----------------------------

MODEL INFO:
Observations: 60
Dependent Variable: dice
Type: Mixed effects linear regression

MODEL FIT:
AIC = -103.85357, BIC = -95.47619
Pseudo-R² (fixed effects) = 0.00000
Pseudo-R² (total) = 0.82638

FIXED EFFECTS:
--------------------------------------------------------------------
                       Est.      S.E.     t val.      d.f.         p
----------------- --------- --------- ---------- --------- ---------
(Intercept)         0.71478   0.04241   16.85342   4.48157   0.00003
--------------------------------------------------------------------

p values calculated using Kenward-Roger standard errors and d.f.

RANDOM EFFECTS:
------------------------------------
  Group      Parameter    Std. Dev.
---------- ------------- -----------
   ptID     (Intercept)    0.11110
   obs      (Intercept)    0.05813
 Residual                  0.05747
------------------------------------

Grouping variables:
----------------------------
 Group   # groups     ICC
------- ---------- ---------
 ptID       20      0.64874
  obs       3       0.17764
----------------------------

Sigmoidcolon -------------------------------------------------

MODEL INFO:
Observations: 113
Dependent Variable: dice
Type: Mixed effects linear regression
```

MODEL FIT:
AIC = -154.40235, BIC = -140.76541
Pseudo-R^2 (fixed effects) = 0.01411
Pseudo-R^2 (total) = 0.48610

FIXED EFFECTS:

	Est.	S.E.	t val.	d.f.	p
(Intercept)	0.83291	0.02462	33.82841	19.84247	0.00000
typeCBCTHS	0.03155	0.01801	1.75174	91.09416	0.08318

p values calculated using Kenward-Roger standard errors and d.f.

RANDOM EFFECTS:

Group	Parameter	Std. Dev.
ptID	(Intercept)	0.09167
obs	(Intercept)	0.00001
Residual		0.09565

Grouping variables:

Group	# groups	ICC
ptID	19	0.47875
obs	3	0.00000

MODEL INFO:
Observations: 57
Dependent Variable: dice
Type: Mixed effects linear regression

MODEL FIT:
AIC = -95.19044, BIC = -87.01824
Pseudo-R^2 (fixed effects) = 0.00000
Pseudo-R^2 (total) = 0.78316

FIXED EFFECTS:

	Est.	S.E.	t val.	d.f.	p
(Intercept)	0.86447	0.02986	28.94751	15.53477	0.00000

p values calculated using Kenward-Roger standard errors and d.f.

RANDOM EFFECTS:

Group	Parameter	Std. Dev.

```
---------- ------------- -----------
   ptID     (Intercept)    0.11529
   obs      (Intercept)    0.01943
 Residual                  0.06152
------------------------------------

Grouping variables:
----------------------------
 Group   # groups     ICC
------- ---------- ---------
 ptID       19      0.76153
  obs       3       0.02163
----------------------------

MODEL INFO:
Observations: 56
Dependent Variable: dice
Type: Mixed effects linear regression

MODEL FIT:
AIC = -74.88857, BIC = -66.78717
Pseudo-R² (fixed effects) = 0.00000
Pseudo-R² (total) = 0.62630

FIXED EFFECTS:
---------------------------------------------------------------------
                        Est.      S.E.     t val.       d.f.        p
----------------- --------- --------- ---------- ---------- ---------
(Intercept)         0.83292   0.02675   31.13784   14.13983   0.00000
---------------------------------------------------------------------

p values calculated using Kenward-Roger standard errors and d.f.

RANDOM EFFECTS:
------------------------------------
  Group      Parameter    Std. Dev.
---------- ------------- -----------
   ptID     (Intercept)    0.10625
   obs      (Intercept)    0.00000
 Residual                  0.08207
------------------------------------

Grouping variables:
----------------------------
 Group   # groups     ICC
------- ---------- ---------
 ptID       19      0.62630
  obs       3       0.00000
----------------------------

Prostate ------------------------------------------------

MODEL INFO:
Observations: 120
```

Dependent Variable: dice
Type: Mixed effects linear regression

MODEL FIT:
AIC = -400.60158, BIC = -386.66412
Pseudo-R^2 (fixed effects) = 0.00125
Pseudo-R^2 (total) = 0.62543

FIXED EFFECTS:

	Est.	S.E.	t val.	d.f.	p
(Intercept)	0.85235	0.01070	79.68729	20.96558	0.00000
typeCBCTHS	0.00389	0.00618	0.63066	97.00000	0.52975

p values calculated using Kenward-Roger standard errors and d.f.

RANDOM EFFECTS:

Group	Parameter	Std. Dev.
ptID	(Intercept)	0.04367
obs	(Intercept)	0.00000
Residual		0.03383

Grouping variables:

Group	# groups	ICC
ptID	20	0.62496
obs	3	0.00000

MODEL INFO:
Observations: 60
Dependent Variable: dice
Type: Mixed effects linear regression

MODEL FIT:
AIC = -205.48074, BIC = -197.10336
Pseudo-R^2 (fixed effects) = 0.00000
Pseudo-R^2 (total) = 0.66952

FIXED EFFECTS:

	Est.	S.E.	t val.	d.f.	p
(Intercept)	0.85624	0.00959	89.24521	15.83837	0.00000

p values calculated using Kenward-Roger standard errors and d.f.

RANDOM EFFECTS:

Group	Parameter	Std. Dev.
ptID	(Intercept)	0.03976
obs	(Intercept)	0.00000
Residual		0.02793

Grouping variables:

Group	# groups	ICC
ptID	20	0.66952
obs	3	0.00000

MODEL INFO:
Observations: 60
Dependent Variable: dice
Type: Mixed effects linear regression

MODEL FIT:
AIC = -184.12040, BIC = -175.74302
Pseudo-R^2 (fixed effects) = 0.00000
Pseudo-R^2 (total) = 0.72168

FIXED EFFECTS:

	Est.	S.E.	t val.	d.f.	p
(Intercept)	0.85235	0.01237	68.90950	16.81786	0.00000

p values calculated using Kenward-Roger standard errors and d.f.

RANDOM EFFECTS:

Group	Parameter	Std. Dev.
ptID	(Intercept)	0.05207
obs	(Intercept)	0.00000
Residual		0.03234

Grouping variables:

Group	# groups	ICC
ptID	20	0.72168
obs	3	0.00000

Rectum --

MODEL INFO:
Observations: 120
Dependent Variable: dice
Type: Mixed effects linear regression

MODEL FIT:
AIC = -344.82806, BIC = -330.89060
Pseudo-R^2 (fixed effects) = 0.00071
Pseudo-R^2 (total) = 0.64946

FIXED EFFECTS:

	Est.	S.E.	t val.	d.f.	p
(Intercept)	0.89115	0.01795	49.63540	6.88467	0.00000
typeCBCTHS	-0.00377	0.00769	-0.49054	97.00000	0.62486

p values calculated using Kenward-Roger standard errors and d.f.

RANDOM EFFECTS:

Group	Parameter	Std. Dev.
ptID	(Intercept)	0.05318
obs	(Intercept)	0.02131
Residual		0.04211

Grouping variables:

Group	# groups	ICC
ptID	20	0.55936
obs	3	0.08986

MODEL INFO:
Observations: 60
Dependent Variable: dice
Type: Mixed effects linear regression

MODEL FIT:
AIC = -185.05468, BIC = -176.67730
Pseudo-R^2 (fixed effects) = 0.00000
Pseudo-R^2 (total) = 0.86578

FIXED EFFECTS:

	Est.	S.E.	t val.	d.f.	p
(Intercept)	0.88737	0.01982	44.76189	8.26610	0.00000

```
p values calculated using Kenward-Roger standard errors and d.f.

RANDOM EFFECTS:
------------------------------------
  Group      Parameter    Std. Dev.
---------- ------------- -----------
   ptID     (Intercept)    0.06546
   obs      (Intercept)    0.02234
 Residual                  0.02723
------------------------------------

Grouping variables:
----------------------------
 Group   # groups     ICC
------- ---------- ---------
 ptID       20      0.77544
  obs       3       0.09034
----------------------------

MODEL INFO:
Observations: 60
Dependent Variable: dice
Type: Mixed effects linear regression

MODEL FIT:
AIC = -151.91954, BIC = -143.54216
Pseudo-R² (fixed effects) = 0.00000
Pseudo-R² (total) = 0.49445

FIXED EFFECTS:
--------------------------------------------------------------------
                       Est.      S.E.     t val.      d.f.         p
----------------- --------- --------- ---------- --------- ---------
(Intercept)         0.89115   0.01632   54.59174   4.46417   0.00000
--------------------------------------------------------------------

p values calculated using Kenward-Roger standard errors and d.f.

RANDOM EFFECTS:
------------------------------------
  Group      Parameter    Std. Dev.
---------- ------------- -----------
   ptID     (Intercept)    0.04326
   obs      (Intercept)    0.02006
 Residual                  0.04822
------------------------------------

Grouping variables:
----------------------------
 Group   # groups     ICC
------- ---------- ---------
 ptID       20      0.40696
  obs       3       0.08749
```

All results show that the ICC's are relatively close to zero, which indicates that the observers are consistent among each other. However, these are point-estimates and confidence intervals have to be constructed in order to perform formal comparison between the HS and cCBCT model. More on this in the next section.

Bootstrapping procedure

A stratified bootstrap method is performed, taking twenty patients from both scans with replacement. This is performed 10.000 times and results for the Intraclass correlation are stored over these iterations. This will allow us to investigate the asymptotic behavior of the ICC coefficients. The Welch Two Sample t-test tests the null-hypothesis of equivalent ICC between the scans. In case this is rejected, there is sufficient evidence to state that the observers were more consistent for one scan, compared to the other. Furthermore, the average and standard deviation of the bootstraps is given below:

```
booted_data = function(data_hs, organi){

  data_hs = subset(data_hs, organ == organi)
  boots = sample(unique(data_hs$ptID), 20, replace = TRUE)

  booted = data_hs[which(data_hs$ptID == boots[1]),]
  for(i in 2:length(boots)){
    booted = rbind(booted, data_hs[which(data_hs$ptID == boots[i]),])
  }
  return(booted)
}


for(organi in unique(data_hs$organ)){


#Print title
cat('\n')
cat(paste(organi, '--------------------------------------------- \n'))
cat('\n')

straps = 10000
iccHS = numeric(straps)
iccCBCT = numeric(straps)

for(j in 1:straps){

  boots = booted_data(data_hs, organi)

  #Fit the models
  fm_A <- lmer(dice ~  (1 | obs) + (1 | ptID) , data = boots, subset = typeCBCT == "HS")
  fm_B <- lmer(dice ~  (1 | obs) + (1 | ptID) , data = boots, subset = typeCBCT == "cCBCT")

  # Extract variance components
  var_A <- as.data.frame(VarCorr(fm_A))
  var_B <- as.data.frame(VarCorr(fm_B))

  # Calculate ICC for each group
  icc_A <- var_A[2, "vcov"] / (var_A[1, "vcov"] + var_A[2, "vcov"] + var_A[3, "vcov"])
  icc_B <- var_B[2, "vcov"] / (var_B[1, "vcov"] + var_B[2, "vcov"] + var_A[3, "vcov"])


  iccHS[j] = icc_A
  iccCBCT[j] = icc_B
```

```r
}

print(t.test(iccCBCT, iccHS))
}
```

```
Bladder ----------------------------------------------------


    Welch Two Sample t-test

data:  iccCBCT and iccHS
t = 1.1506, df = 14983, p-value = 0.2499
alternative hypothesis: true difference in means is not equal to 0
95 percent confidence interval:
 -0.001566336  0.006018605
sample estimates:
 mean of x  mean of y
0.10131811 0.09909198


Seminalvesicle ---------------------------------------------


    Welch Two Sample t-test

data:  iccCBCT and iccHS
t = 67.74, df = 19564, p-value < 2.2e-16
alternative hypothesis: true difference in means is not equal to 0
95 percent confidence interval:
 0.07778993 0.08242582
sample estimates:
mean of x mean of y
0.2169384 0.1368305


Sigmoidcolon -----------------------------------------------


    Welch Two Sample t-test

data:  iccCBCT and iccHS
t = -42.848, df = 19761, p-value < 2.2e-16
alternative hypothesis: true difference in means is not equal to 0
95 percent confidence interval:
 -0.02188643 -0.01997165
sample estimates:
 mean of x  mean of y
0.01822879 0.03915783


Prostate ---------------------------------------------------
```

```
    Welch Two Sample t-test

data:  iccCBCT and iccHS
t = -10.048, df = 19883, p-value < 2.2e-16
alternative hypothesis: true difference in means is not equal to 0
95 percent confidence interval:
 -0.003575961 -0.002408586
sample estimates:
 mean of x  mean of y 
0.01051243 0.01350470 


Rectum --------------------------------------------


    Welch Two Sample t-test

data:  iccCBCT and iccHS
t = 17.254, df = 18002, p-value < 2.2e-16
alternative hypothesis: true difference in means is not equal to 0
95 percent confidence interval:
 0.01796897 0.02257497
sample estimates:
mean of x mean of y 
0.1534125 0.1331406 
```

The Sigmoid colon, and the Prostate show a significant results in favor of the conventional CBCT (cCBCT), whereas the Rectum and the Seminal Vesicle show a significant results in favor of the high-performance CBCT (HS). The Bladder does not show any statistically significant difference between the two scans. Note, that the absolute difference between the ICC's that are already very close to zero, is very small. If this is difference is to be considered clinically relevant is up for expert discussions.

Hausdorff Distance

The same procedure is repeated for the 95th percentile Hausdorff distance (VSDP)

Effect CBCT type on Autosegmentation performance

```
library(lme4)

for(organi in unique(data_hs$organ)){

#Print title
cat('\n')
cat(paste(organi, '------------------------------------------------ \n'))
cat('\n')

#Fit the regression model with random effects for the observers and patients
fm = lmer(vsdp ~ typeCBCT  + (1 | obs) + (1 | ptID) , data = data_hs, subset = organ == organi)
print(summ(fm, digits = 5))


#Fit the regression model with random effects only for the observers
nm = lmer(vsdp ~ typeCBCT  + (1 | obs) , data = data_hs, subset = organ == organi)


# Compare most suitable model with the Likelihood-ratio test
library(lmtest)
print(lrtest(nm, fm))
#cat('\n')
print(paste('BIC statistic for the original model : ', round(BIC(fm)) ,  "\n"))
print(paste('BIC statistic for the nested model : ', round(BIC(nm)), "\n"))
}
```

```
Bladder ------------------------------------------------

MODEL INFO:
Observations: 120
Dependent Variable: vsdp
Type: Mixed effects linear regression

MODEL FIT:
AIC = 781.99515, BIC = 795.93261
Pseudo-R² (fixed effects) = 0.00693
Pseudo-R² (total) = 0.84483

FIXED EFFECTS:
----------------------------------------------------------------------
                          Est.      S.E.      t val.        d.f.         p
----------------- ---------- --------- ---------- ---------- ---------
(Intercept)            6.48758   2.48512    2.61057   19.98461   0.01675
typeCBCTHS            -1.95337   0.84744   -2.30502   97.00000   0.02330
----------------------------------------------------------------------
```

```
p values calculated using Kenward-Roger standard errors and d.f.

RANDOM EFFECTS:
------------------------------------
  Group      Parameter    Std. Dev.
---------- ------------- -----------
   ptID     (Intercept)   10.78588
   obs      (Intercept)    0.00000
 Residual                  4.64163
------------------------------------

Grouping variables:
----------------------------
 Group   # groups     ICC
------- ---------- ---------
 ptID       20      0.84374
  obs       3       0.00000
----------------------------
Likelihood ratio test

Model 1: vsdp ~ typeCBCT + (1 | obs)
Model 2: vsdp ~ typeCBCT + (1 | obs) + (1 | ptID)
  #Df  LogLik Df  Chisq Pr(>Chisq)
1   4 -460.47
2   5 -386.00  1 148.95  < 2.2e-16 ***
---
Signif. codes:  0 '***' 0.001 '**' 0.01 '*' 0.05 '.' 0.1 ' ' 1
[1] "BIC statistic for the original model :  796 \n"
[1] "BIC statistic for the nested model :  940 \n"

Seminalvesicle ---------------------------------------------

MODEL INFO:
Observations: 120
Dependent Variable: vsdp
Type: Mixed effects linear regression

MODEL FIT:
AIC = 591.89993, BIC = 605.83739
Pseudo-R² (fixed effects) = 0.00582
Pseudo-R² (total) = 0.72387

FIXED EFFECTS:
----------------------------------------------------------------------
                        Est.      S.E.      t val.       d.f.        p
----------------- ---------- ---------- ---------- ---------- ---------
(Intercept)          6.76326   0.91139     7.42084   16.66927   0.00000
typeCBCTHS          -0.62779   0.39630    -1.58413   97.00000   0.11642
----------------------------------------------------------------------

p values calculated using Kenward-Roger standard errors and d.f.

RANDOM EFFECTS:
```

```
-------------------------------------
  Group      Parameter    Std. Dev.
---------- ------------- -----------
   ptID     (Intercept)    3.42922
   obs      (Intercept)    0.70169
 Residual                  2.17061
-------------------------------------

Grouping variables:
----------------------------
 Group   # groups     ICC
------- ---------- ---------
 ptID       20      0.69323
  obs       3       0.02903
----------------------------
Likelihood ratio test

Model 1: vsdp ~ typeCBCT + (1 | obs)
Model 2: vsdp ~ typeCBCT + (1 | obs) + (1 | ptID)
  #Df  LogLik Df  Chisq Pr(>Chisq)
1   4 -336.50
2   5 -290.95  1 91.099  < 2.2e-16 ***
---
Signif. codes:  0 '***' 0.001 '**' 0.01 '*' 0.05 '.' 0.1 ' ' 1
[1] "BIC statistic for the original model :  606 \n"
[1] "BIC statistic for the nested model :  692 \n"

Sigmoidcolon -------------------------------------------

MODEL INFO:
Observations: 113
Dependent Variable: vsdp
Type: Mixed effects linear regression

MODEL FIT:
AIC = 946.35917, BIC = 959.99611
Pseudo-R² (fixed effects) = 0.00043
Pseudo-R² (total) = 0.20614

FIXED EFFECTS:
----------------------------------------------------------------------
                         Est.      S.E.     t val.       d.f.        p
----------------- ---------- --------- ---------- ---------- ---------
(Intercept)         12.00803   2.92062    4.11146    8.05494   0.00333
typeCBCTHS          -0.68146   2.76265   -0.24667   91.15920   0.80572
----------------------------------------------------------------------

p values calculated using Kenward-Roger standard errors and d.f.

RANDOM EFFECTS:
-------------------------------------
  Group      Parameter    Std. Dev.
---------- ------------- -----------
   ptID     (Intercept)    7.04235
```

```
   obs      (Intercept)    2.48877
 Residual                 14.67295
------------------------------------

Grouping variables:
----------------------------
 Group   # groups     ICC
------- ---------- ---------
 ptID       19      0.18295
  obs       3       0.02285
----------------------------
Likelihood ratio test

Model 1: vsdp ~ typeCBCT + (1 | obs)
Model 2: vsdp ~ typeCBCT + (1 | obs) + (1 | ptID)
  #Df  LogLik Df  Chisq Pr(>Chisq)
1   4 -471.54
2   5 -468.18  1 6.7178   0.009546 **
---
Signif. codes:  0 '***' 0.001 '**' 0.01 '*' 0.05 '.' 0.1 ' ' 1
[1] "BIC statistic for the original model :  960 \n"
[1] "BIC statistic for the nested model :  962 \n"

Prostate ---------------------------------------------

MODEL INFO:
Observations: 120
Dependent Variable: vsdp
Type: Mixed effects linear regression

MODEL FIT:
AIC = 421.38051, BIC = 435.31797
Pseudo-R² (fixed effects) = 0.01242
Pseudo-R² (total) = 0.44508

FIXED EFFECTS:
----------------------------------------------------------------------
                        Est.      S.E.     t val.      d.f.          p
----------------- ---------- --------- ---------- ---------- ---------
(Intercept)          5.37378   0.28069   19.14510   18.53896   0.00000
typeCBCTHS          -0.34536   0.21161   -1.63203   97.00000   0.10591
----------------------------------------------------------------------

p values calculated using Kenward-Roger standard errors and d.f.

RANDOM EFFECTS:
------------------------------------
  Group      Parameter    Std. Dev.
---------- ------------- -----------
   ptID     (Intercept)    1.01647
   obs      (Intercept)    0.11918
 Residual                  1.15906
------------------------------------
```

```
Grouping variables:
----------------------------
 Group   # groups     ICC
------- ---------- ---------
 ptID       20      0.43216
  obs       3       0.00594
----------------------------
Likelihood ratio test

Model 1: vsdp ~ typeCBCT + (1 | obs)
Model 2: vsdp ~ typeCBCT + (1 | obs) + (1 | ptID)
  #Df  LogLik Df Chisq Pr(>Chisq)
1   4 -221.97
2   5 -205.69  1 32.56  1.156e-08 ***
---
Signif. codes:  0 '***' 0.001 '**' 0.01 '*' 0.05 '.' 0.1 ' ' 1
[1] "BIC statistic for the original model :  435 \n"
[1] "BIC statistic for the nested model :  463 \n"

Rectum ---------------------------------------------

MODEL INFO:
Observations: 120
Dependent Variable: vsdp
Type: Mixed effects linear regression

MODEL FIT:
AIC = 767.08922, BIC = 781.02668
Pseudo-R² (fixed effects) = 0.00009
Pseudo-R² (total) = 0.49506

FIXED EFFECTS:
----------------------------------------------------------------------
                          Est.       S.E.     t val.       d.f.         p
----------------- ---------- ---------- ---------- ---------- ---------
(Intercept)            8.37677    1.26820    6.60525   20.75883   0.00000
typeCBCTHS            -0.13037    0.90346   -0.14430   97.00000   0.88556
----------------------------------------------------------------------

p values calculated using Kenward-Roger standard errors and d.f.

RANDOM EFFECTS:
------------------------------------
  Group      Parameter    Std. Dev.
---------- ------------- -----------
   ptID     (Intercept)    4.89940
   obs      (Intercept)    0.00000
 Residual                  4.94848
------------------------------------

Grouping variables:
----------------------------
 Group   # groups     ICC
------- ---------- ---------
```

```
 ptID       20      0.49502
  obs       3       0.00000
----------------------------
Likelihood ratio test

Model 1: vsdp ~ typeCBCT + (1 | obs)
Model 2: vsdp ~ typeCBCT + (1 | obs) + (1 | ptID)
  #Df  LogLik Df  Chisq Pr(>Chisq)
1   4 -399.53
2   5 -378.54  1 41.975  9.243e-11 ***
---
Signif. codes:  0 '***' 0.001 '**' 0.01 '*' 0.05 '.' 0.1 ' ' 1
[1] "BIC statistic for the original model :  781 \n"
[1] "BIC statistic for the nested model :  818 \n"
```

ICC: Intra Class Correlation

```
for(organi in unique(data_hs$organ)){
  #Print title
  cat('\n')
  cat(paste(organi, '--------------------------------------------- \n'))
  cat('\n')

  #Calculate ICC for full model
  data_hsi = subset(data_hs, organ == organi)
  fm = lmer(vsdp ~ typeCBCT  + (1 | obs) + (1 | ptID) , data = data_hsi)
  print(summ(fm, digits = 5))

  #Calculate ICC for Hypersight records
  cat('\n')
  fm = lmer(vsdp ~ (1 | obs) + (1 | ptID) , data = data_hsi, subset = typeCBCT == "HS")
  print(summ(fm, digits = 5))

  #Calculate ICC for cCBCT records
  cat('\n')
  fm = lmer(vsdp ~ (1 | obs) + (1 | ptID) , data = data_hsi, subset = typeCBCT == "cCBCT")
  print(summ(fm, digits = 5))

}
```

```
Bladder ---------------------------------------------

MODEL INFO:
Observations: 120
Dependent Variable: vsdp
Type: Mixed effects linear regression

MODEL FIT:
AIC = 781.99515, BIC = 795.93261
Pseudo-R² (fixed effects) = 0.00693
Pseudo-R² (total) = 0.84483
```

FIXED EFFECTS:

	Est.	S.E.	t val.	d.f.	p
(Intercept)	6.48758	2.48512	2.61057	19.98461	0.01675
typeCBCTHS	-1.95337	0.84744	-2.30502	97.00000	0.02330

p values calculated using Kenward-Roger standard errors and d.f.

RANDOM EFFECTS:

Group	Parameter	Std. Dev.
ptID	(Intercept)	10.78588
obs	(Intercept)	0.00000
Residual		4.64163

Grouping variables:

Group	# groups	ICC
ptID	20	0.84374
obs	3	0.00000

MODEL INFO:
Observations: 60
Dependent Variable: vsdp
Type: Mixed effects linear regression

MODEL FIT:
AIC = 243.27075, BIC = 251.64813
Pseudo-R^2 (fixed effects) = 0.00000
Pseudo-R^2 (total) = 0.99164

FIXED EFFECTS:

	Est.	S.E.	t val.	d.f.	p
(Intercept)	4.53421	1.57991	2.86991	19.82704	0.00952

p values calculated using Kenward-Roger standard errors and d.f.

RANDOM EFFECTS:

Group	Parameter	Std. Dev.
ptID	(Intercept)	6.96776
obs	(Intercept)	0.43057
Residual		0.64111

```
------------------------------------

Grouping variables:
----------------------------
 Group   # groups     ICC
------- ---------- ---------
 ptID       20      0.98786
  obs       3       0.00377
----------------------------

MODEL INFO:
Observations: 60
Dependent Variable: vsdp
Type: Mixed effects linear regression

MODEL FIT:
AIC = 369.00291, BIC = 377.38029
Pseudo-R² (fixed effects) = 0.00000
Pseudo-R² (total) = 0.97731

FIXED EFFECTS:
--------------------------------------------------------------------
                       Est.      S.E.    t val.       d.f.         p
----------------- --------- --------- --------- ---------- ---------
(Intercept)         6.48758   3.35065   1.93622   18.98880   0.06787
--------------------------------------------------------------------

p values calculated using Kenward-Roger standard errors and d.f.

RANDOM EFFECTS:
------------------------------------
  Group      Parameter    Std. Dev.
---------- ------------- -----------
   ptID     (Intercept)   14.92691
   obs      (Intercept)    0.00000
 Residual                  2.27429
------------------------------------

Grouping variables:
----------------------------
 Group   # groups     ICC
------- ---------- ---------
 ptID       20      0.97731
  obs       3       0.00000
----------------------------

Seminalvesicle ---------------------------------------------

MODEL INFO:
Observations: 120
Dependent Variable: vsdp
Type: Mixed effects linear regression

MODEL FIT:
```

AIC = 591.89993, BIC = 605.83739
Pseudo-R^2 (fixed effects) = 0.00582
Pseudo-R^2 (total) = 0.72387

FIXED EFFECTS:

	Est.	S.E.	t val.	d.f.	p
(Intercept)	6.76326	0.91139	7.42084	16.66927	0.00000
typeCBCTHS	-0.62779	0.39630	-1.58413	97.00000	0.11642

p values calculated using Kenward-Roger standard errors and d.f.

RANDOM EFFECTS:

Group	Parameter	Std. Dev.
ptID	(Intercept)	3.42922
obs	(Intercept)	0.70169
Residual		2.17061

Grouping variables:

Group	# groups	ICC
ptID	20	0.69323
obs	3	0.02903

MODEL INFO:
Observations: 60
Dependent Variable: vsdp
Type: Mixed effects linear regression

MODEL FIT:
AIC = 278.98693, BIC = 287.36431
Pseudo-R^2 (fixed effects) = 0.00000
Pseudo-R^2 (total) = 0.89668

FIXED EFFECTS:

	Est.	S.E.	t val.	d.f.	p
(Intercept)	6.13547	0.94409	6.49879	19.14279	0.00000

p values calculated using Kenward-Roger standard errors and d.f.

RANDOM EFFECTS:

Group	Parameter	Std. Dev.

```
   ptID     (Intercept)    3.89720
   obs      (Intercept)    0.55357
 Residual                  1.33619
------------------------------------

Grouping variables:
----------------------------
 Group   # groups     ICC
------- ---------- ---------
 ptID       20      0.87894
  obs       3       0.01773
----------------------------

MODEL INFO:
Observations: 60
Dependent Variable: vsdp
Type: Mixed effects linear regression

MODEL FIT:
AIC = 301.84604, BIC = 310.22342
Pseudo-R² (fixed effects) = 0.00000
Pseudo-R² (total) = 0.80170

FIXED EFFECTS:
--------------------------------------------------------------------
                       Est.      S.E.    t val.       d.f.         p
----------------- ---------- ---------- ---------- ---------- ----------
(Intercept)         6.76326   0.96420   7.01437   12.73587   0.00001
--------------------------------------------------------------------

p values calculated using Kenward-Roger standard errors and d.f.

RANDOM EFFECTS:
------------------------------------
  Group      Parameter    Std. Dev.
---------- ------------- -----------
   ptID     (Intercept)    3.57304
   obs      (Intercept)    0.84107
 Residual                  1.82562
------------------------------------

Grouping variables:
----------------------------
 Group   # groups     ICC
------- ---------- ---------
 ptID       20      0.75961
  obs       3       0.04209
----------------------------

Sigmoidcolon -----------------------------------------------

MODEL INFO:
Observations: 113
Dependent Variable: vsdp
```

Type: Mixed effects linear regression

MODEL FIT:
AIC = 946.35917, BIC = 959.99611
Pseudo-R^2 (fixed effects) = 0.00043
Pseudo-R^2 (total) = 0.20614

FIXED EFFECTS:

	Est.	S.E.	t val.	d.f.	p
(Intercept)	12.00803	2.92062	4.11146	8.05494	0.00333
typeCBCTHS	-0.68146	2.76265	-0.24667	91.15920	0.80572

p values calculated using Kenward-Roger standard errors and d.f.

RANDOM EFFECTS:

Group	Parameter	Std. Dev.
ptID	(Intercept)	7.04235
obs	(Intercept)	2.48877
Residual		14.67295

Grouping variables:

Group	# groups	ICC
ptID	19	0.18295
obs	3	0.02285

MODEL INFO:
Observations: 57
Dependent Variable: vsdp
Type: Mixed effects linear regression

MODEL FIT:
AIC = 492.23908, BIC = 500.41129
Pseudo-R^2 (fixed effects) = 0.00000
Pseudo-R^2 (total) = 0.39735

FIXED EFFECTS:

	Est.	S.E.	t val.	d.f.	p
(Intercept)	11.32657	3.51291	3.22427	7.71669	0.01276

p values calculated using Kenward-Roger standard errors and d.f.

RANDOM EFFECTS:

```
------------------------------------
  Group      Parameter    Std. Dev.
---------- ------------- -----------
   ptID     (Intercept)   11.82758
   obs      (Intercept)    1.86762
 Residual                 14.74663
------------------------------------

Grouping variables:
----------------------------
 Group   # groups     ICC
------- ---------- ---------
 ptID       19      0.38768
  obs       3       0.00967
----------------------------

MODEL INFO:
Observations: 56
Dependent Variable: vsdp
Type: Mixed effects linear regression

MODEL FIT:
AIC = 449.99480, BIC = 458.09621
Pseudo-R² (fixed effects) = 0.00000
Pseudo-R² (total) = 0.26500

FIXED EFFECTS:
------------------------------------------------------------------
                        Est.      S.E.    t val.      d.f.         p
----------------- ---------- --------- --------- --------- ---------
(Intercept)         12.05688   2.33128   5.17179   4.92581   0.00371
------------------------------------------------------------------

p values calculated using Kenward-Roger standard errors and d.f.

RANDOM EFFECTS:
------------------------------------
  Group      Parameter    Std. Dev.
---------- ------------- -----------
   ptID     (Intercept)    6.77202
   obs      (Intercept)    1.38562
 Residual                 11.51180
------------------------------------

Grouping variables:
----------------------------
 Group   # groups     ICC
------- ---------- ---------
 ptID       19      0.25435
  obs       3       0.01065
----------------------------

Prostate ---------------------------------------------
```

MODEL INFO:
Observations: 120
Dependent Variable: vsdp
Type: Mixed effects linear regression

MODEL FIT:
AIC = 421.38051, BIC = 435.31797
Pseudo-R^2 (fixed effects) = 0.01242
Pseudo-R^2 (total) = 0.44508

FIXED EFFECTS:

	Est.	S.E.	t val.	d.f.	p
(Intercept)	5.37378	0.28069	19.14510	18.53896	0.00000
typeCBCTHS	-0.34536	0.21161	-1.63203	97.00000	0.10591

p values calculated using Kenward-Roger standard errors and d.f.

RANDOM EFFECTS:

Group	Parameter	Std. Dev.
ptID	(Intercept)	1.01647
obs	(Intercept)	0.11918
Residual		1.15906

Grouping variables:

Group	# groups	ICC
ptID	20	0.43216
obs	3	0.00594

MODEL INFO:
Observations: 60
Dependent Variable: vsdp
Type: Mixed effects linear regression

MODEL FIT:
AIC = 196.35458, BIC = 204.73196
Pseudo-R^2 (fixed effects) = 0.00000
Pseudo-R^2 (total) = 0.61033

FIXED EFFECTS:

	Est.	S.E.	t val.	d.f.	p
(Intercept)	5.02842	0.26990	18.63039	14.38620	0.00000

p values calculated using Kenward-Roger standard errors and d.f.

RANDOM EFFECTS:

Group	Parameter	Std. Dev.
ptID	(Intercept)	1.08815
obs	(Intercept)	0.05512
Residual		0.87059

Grouping variables:

Group	# groups	ICC
ptID	20	0.60877
obs	3	0.00156

MODEL INFO:
Observations: 60
Dependent Variable: vsdp
Type: Mixed effects linear regression

MODEL FIT:
AIC = 225.75482, BIC = 234.13220
Pseudo-R^2 (fixed effects) = 0.00000
Pseudo-R^2 (total) = 0.51563

FIXED EFFECTS:

	Est.	S.E.	t val.	d.f.	p
(Intercept)	5.37378	0.31053	17.30527	12.11245	0.00000

p values calculated using Kenward-Roger standard errors and d.f.

RANDOM EFFECTS:

Group	Parameter	Std. Dev.
ptID	(Intercept)	1.21189
obs	(Intercept)	0.00000
Residual		1.17459

Grouping variables:

Group	# groups	ICC
ptID	20	0.51563
obs	3	0.00000

Rectum ---

MODEL INFO:
Observations: 120
Dependent Variable: vsdp
Type: Mixed effects linear regression

MODEL FIT:
AIC = 767.08922, BIC = 781.02668
Pseudo-R^2 (fixed effects) = 0.00009
Pseudo-R^2 (total) = 0.49506

FIXED EFFECTS:

	Est.	S.E.	t val.	d.f.	p
(Intercept)	8.37677	1.26820	6.60525	20.75883	0.00000
typeCBCTHS	-0.13037	0.90346	-0.14430	97.00000	0.88556

p values calculated using Kenward-Roger standard errors and d.f.

RANDOM EFFECTS:

Group	Parameter	Std. Dev.
ptID	(Intercept)	4.89940
obs	(Intercept)	0.00000
Residual		4.94848

Grouping variables:

Group	# groups	ICC
ptID	20	0.49502
obs	3	0.00000

MODEL INFO:
Observations: 60
Dependent Variable: vsdp
Type: Mixed effects linear regression

MODEL FIT:
AIC = 361.01791, BIC = 369.39529
Pseudo-R^2 (fixed effects) = 0.00000
Pseudo-R^2 (total) = 0.77781

FIXED EFFECTS:

	Est.	S.E.	t val.	d.f.	p

```
(Intercept)         8.24640   1.37554   5.99502   17.66480   0.00001
--------------------------------------------------------------------

p values calculated using Kenward-Roger standard errors and d.f.

RANDOM EFFECTS:
------------------------------------
  Group      Parameter    Std. Dev.
---------- ------------- -----------
   ptID     (Intercept)    5.87812
   obs      (Intercept)    0.00004
 Residual                  3.14166
------------------------------------

Grouping variables:
----------------------------
 Group   # groups     ICC
------- ---------- ---------
 ptID       20      0.77781
  obs       3       0.00000
----------------------------

MODEL INFO:
Observations: 60
Dependent Variable: vsdp
Type: Mixed effects linear regression

MODEL FIT:
AIC = 406.70767, BIC = 415.08505
Pseudo-R² (fixed effects) = 0.00000
Pseudo-R² (total) = 0.33949

FIXED EFFECTS:
-------------------------------------------------------------------
                       Est.      S.E.    t val.      d.f.         p
----------------- --------- --------- --------- --------- ---------
(Intercept)         8.37677   1.21448   6.89740   7.45793   0.00017
-------------------------------------------------------------------

p values calculated using Kenward-Roger standard errors and d.f.

RANDOM EFFECTS:
------------------------------------
  Group      Parameter    Std. Dev.
---------- ------------- -----------
   ptID     (Intercept)    4.23017
   obs      (Intercept)    0.00000
 Residual                  5.90042
------------------------------------

Grouping variables:
----------------------------
 Group   # groups     ICC
------- ---------- ---------
```

```
 ptID       20       0.33949
  obs       3        0.00000
----------------------------
```

Bootstrapping procedure

```
for(organi in unique(data_hs$organ)){

cat(paste0("### Organ : ", organi, "\n"))

#Print title
cat('\n')
cat(paste(organi, '--------------------------------------------- \n'))
cat('\n')

straps = 10000
iccHS = numeric(straps)
iccCBCT = numeric(straps)

for(j in 1:straps){

  boots = booted_data(data_hs, organi)

  #Fit the models
  fm_A <- lmer(vsdp ~  (1 | obs) + (1 | ptID) , data = boots, subset = typeCBCT == "HS")
  fm_B <- lmer(vsdp ~  (1 | obs) + (1 | ptID) , data = boots, subset = typeCBCT == "cCBCT")

  # Extract variance components
  var_A <- as.data.frame(VarCorr(fm_A))
  var_B <- as.data.frame(VarCorr(fm_B))

  # Calculate ICC for each group
  icc_A <- var_A[2, "vcov"] / (var_A[1, "vcov"] + var_A[2, "vcov"] + var_A[3, "vcov"])
  icc_B <- var_B[2, "vcov"] / (var_B[1, "vcov"] + var_B[2, "vcov"] + var_A[3, "vcov"])


  iccHS[j] = icc_A
  iccCBCT[j] = icc_B

}

print(t.test(iccCBCT, iccHS))
}
```

```
### Organ : Bladder

Bladder ---------------------------------------------


    Welch Two Sample t-test

data:  iccCBCT and iccHS
```

```
t = -43.66, df = 12538, p-value < 2.2e-16
alternative hypothesis: true difference in means is not equal to 0
95 percent confidence interval:
 -0.04623541 -0.04226224
sample estimates:
 mean of x  mean of y
0.01652767 0.06077650
```

```
### Organ : Seminalvesicle

Seminalvesicle ---------------------------------------------


    Welch Two Sample t-test

data:  iccCBCT and iccHS
t = 43.437, df = 16210, p-value < 2.2e-16
alternative hypothesis: true difference in means is not equal to 0
95 percent confidence interval:
 0.03407589 0.03729662
sample estimates:
 mean of x  mean of y
0.07455798 0.03887173

### Organ : Sigmoidcolon

Sigmoidcolon ----------------------------------------------


    Welch Two Sample t-test

data:  iccCBCT and iccHS
t = 6.0053, df = 17178, p-value = 1.949e-09
alternative hypothesis: true difference in means is not equal to 0
95 percent confidence interval:
 0.002858709 0.005629112
sample estimates:
 mean of x  mean of y
0.04389617 0.03965226

### Organ : Prostate

Prostate ------------------------------------------------


    Welch Two Sample t-test

data:  iccCBCT and iccHS
t = 11.006, df = 19675, p-value < 2.2e-16
alternative hypothesis: true difference in means is not equal to 0
95 percent confidence interval:
 0.004394126 0.006298276
sample estimates:
mean of x mean of y
```

```
0.0319035 0.0265573

### Organ : Rectum

Rectum ---------------------------------------------


    Welch Two Sample t-test

data:  iccCBCT and iccHS
t = 42.664, df = 13370, p-value < 2.2e-16
alternative hypothesis: true difference in means is not equal to 0
95 percent confidence interval:
 0.02487282 0.02726836
sample estimates:
mean of x  mean of y

0.03812254 0.01205196
```

The significant t-test for the Rectum, Sigmoid colon, and Seminal Vesicle demonstrates that evidence in improvement in intra-observer consistency with the high-performance CBCT (HS). The conventional CBCT (cCBCT) gives statistically significant results in the consistency among observers for the Bladder and the Prostate. Note, that the clinical significance of the small difference in ICC's is up for expert discussions.

Note

This file version (4) may be subject to change. All documentation and code is solely intended for the purpose of this research, please ask the author for permission in case of sharing (*a.elyaakoubi@erasmusmc.nl*).
